# 5-HT_1F_ receptor agonism induces mitochondrial biogenesis and increases cellular function in brain microvascular endothelial cells

**DOI:** 10.3389/fncel.2024.1365158

**Published:** 2024-03-05

**Authors:** Natalie E. Scholpa, Epiphani C. Simmons, Austin D. Thompson, Seth S. Carroll, Rick G. Schnellmann

**Affiliations:** ^1^Department of Pharmacology and Toxicology, College of Pharmacy, University of Arizona, Tucson, AZ, United States; ^2^Southern Arizona VA Health Care System, Tucson, AZ, United States; ^3^Department of Neurosciences, College of Medicine, University of Arizona, Tucson, AZ, United States; ^4^Southwest Environmental Health Science Center, University of Arizona, Tucson, AZ, United States; ^5^Center for Innovation in Brain Science, University of Arizona, Tucson, AZ, United States

**Keywords:** mitochondrial biogenesis, lasmiditan, 5-HT_1F_ receptor, endothelial cells, blood–brain barrier, blood–spinal cord barrier, vascular recovery

## Abstract

**Introduction:**

Vascular and mitochondrial dysfunction are well-established consequences of multiple central nervous system (CNS) disorders, including neurodegenerative diseases and traumatic injuries. We previously reported that 5-hydroxytryptamine 1F receptor (5-HT_1F_R) agonism induces mitochondrial biogenesis (MB) in multiple organ systems, including the CNS.

**Methods:**

Lasmiditan is a selective 5-HT_1F_R agonist that is FDA-approved for the treatment of migraines. We have recently shown that lasmiditan treatment induces MB, promotes vascular recovery and improves locomotor function in a mouse model of spinal cord injury (SCI). To investigate the mechanism of this effect, primary cerebral microvascular endothelial cells from C57bl/6 mice (mBMEC) were used.

**Results:**

Lasmiditan treatment increased the maximal oxygen consumption rate, mitochondrial proteins and mitochondrial density in mBMEC, indicative of MB induction. Lasmiditan also enhanced endothelial cell migration and tube formation, key components of angiogenesis. Trans-endothelial electrical resistance (TEER) and tight junction protein expression, including claudin-5, were also increased with lasmiditan, suggesting improved barrier function. Finally, lasmiditan treatment decreased phosphorylated VE-Cadherin and induced activation of the Akt-FoxO1 pathway, which decreases FoxO1-mediated inhibition of claudin-5 transcription.

**Discussion:**

These data demonstrate that lasmiditan induces MB and enhances endothelial cell function, likely via the VE-Cadherin-Akt-FoxO1-claudin-5 signaling axis. Given the importance of mitochondrial and vascular dysfunction in neuropathologies, 5-HT_1F_R agonism may have broad therapeutic potential to address multiple facets of disease progression by promoting MB and vascular recovery.

## 1 Introduction

Disruption of mitochondrial function is implicated in multiple central nervous system (CNS) pathologies, including both acute traumas and neurodegenerative diseases (Simmons et al., 2020b). Because of this, there is continued interest in therapeutic interventions targeting mitochondrial function and homeostasis. The therapeutic potential of inducing mitochondrial biogenesis (MB), the generation of new functioning mitochondria (Cameron et al., 2016; Gibbs et al., 2018c), in the treatment of CNS pathologies remains a promising, though underexplored strategy (Scholpa and Schnellmann, 2017; Simmons et al., 2020b, 2022). Multiple reports demonstrate the efficacy of MB-related therapies in restoring mitochondrial homeostasis and improving outcomes across preclinical models of both traumatic injuries such as spinal cord injury (SCI), stroke, and traumatic brain injury (TBI), as well as neurodegenerative disorders including Alzheimer’s and Parkinson’s diseases (Gibbs et al., 2018c; Simmons et al., 2020b).

Blood–CNS barriers are comprised of endothelial cells, astrocytes, and pericytes, which work concurrently to form a selectively permeable barrier that both prevents blood-borne pathogens and toxins from entering the CNS and aids in maintaining CNS homeostasis (Daneman and Prat, 2015). Traumatic CNS injuries often result in catastrophic destruction of the blood–brain barrier (BBB) and/or blood–spinal cord barrier (BSCB), leading to enhanced permeability and neuroinflammation that further propagates injury progression (Kumar et al., 2018; Walker et al., 2019). Additionally, evidence of endothelial dysfunction and BBB impairment, including decreased tight junction protein expression, has been identified in patients with Alzheimer’s disease (Yamazaki et al., 2019; Wu et al., 2021), Parkinson’s disease (Gray and Woulfe, 2015), Huntington’s disease (Drouin-Ouellet et al., 2015), and amyotrophic lateral sclerosis (ALS), which is also characterized by decreased BSCB integrity (Henkel et al., 2009; Garbuzova-Davis et al., 2012). Furthermore, blood–CNS barrier breakdown occurs naturally with aging, which, while not associated with negative consequences itself, may become more disadvantageous when exposed to a secondary offense, such as inflammation (Knox et al., 2022).

Restoration of blood–CNS barrier integrity has been associated with improved outcomes in both neurodegenerative disorders and traumatic injuries (Sweeney et al., 2018; van Vliet et al., 2020; Jin et al., 2021; Alarcan et al., 2022; Sousa et al., 2023). Reversing or preventing the loss of tight junctions could aid in this restoration and ultimately ameliorate disease symptoms and/or progression (Li et al., 2021). Angiogenesis has been correlated with enhanced recovery following stroke (Yang and Torbey, 2020), TBI (Chodobski et al., 2011), and SCI (Tsivelekas et al., 2022). Importantly, published studies demonstrate a positive correlation between MB induction and angiogenesis (Arany et al., 2008; Saint-Geniez et al., 2013; Thom et al., 2014). We showed that 5-HT_1F_ receptor (5-HT_1F_R) agonism induces MB in multiple organ systems, including the CNS (Garrett et al., 2014; Gibbs et al., 2018a; Scholpa et al., 2018; Dupre et al., 2019; Simmons et al., 2020a, 2021), and promotes angiogenesis in glomerular endothelial cells *in vitro* (Dupre et al., 2019). Treatment with lasmiditan, a highly selective and potent 5-HT_1F_R agonist FDA-approved for the treatment of migraines, not only induces MB in the spinal cord, but also increases tight junction protein expression, improves BSCB integrity, and enhances locomotor recovery in a mouse model of SCI (Simmons et al., 2021). Despite these promising data, the mechanism of this effect remains unknown. To begin to address this gap in knowledge, we assessed the effects of lasmiditan on MB and endothelial cell function in primary brain microvascular endothelial cells (mBMEC).

## 2 Methods

### 2.1 Cell culture and reagents

Primary C57bl/6 mouse cerebral endothelial cells (mBMEC) were purchased from Cell Biologics Inc. (C57-6023; Chicago, IL). mBMEC were cultured on 0.1% gelatin-coated flasks using endothelial cell medium (M1168, Cell Biologics, Inc.) with growth factor supplement additives (Cell Biologics, Inc.) and 5% (v/v) fetal bovine serum (Cell Biologics, Inc.). mBMEC were used at passages 2–5, and at least two different lots were used for each experiment. Mouse cerebellar astrocytes were purchased from ATCC (C8-D1A; Manassas, VA) and cultured in DMEM with 10% fetal bovine serum for trans-endothelial electrical resistance (TEER) experiments. Lasmiditan was purchased from Tocris (Ellisville, MO).

### 2.2 PCR

DNA was isolated from mBMEC, wild-type C57bl/6NJ spinal cord tissue, and 5-HT_1F_ receptor knockout mouse (B6N(Cg)-*Htr1f^tm1.1(KOMP)Vlcg^*/J; Jackson Laboratories) spinal cord tissue using the Qiagen DNeasy Blood and Tissue Kit (Valencia, CA). To determine the presence of the 5-HT_1F_R gene, DNA was amplified using Promega 2x PCR Master Mix (Promega, Madison, WI) in accordance with the manufacturer’s protocols. Amplified DNA was separated on a 1.5% agarose gel and visualized by ethidium bromide fluorescence. Primers used were sense: 5′-GCCGTGATGATGAGTGTGTC-3′ and antisense: 5′-ACTATCCGACTCGCTTGTCT-′3.

### 2.3 Analysis of oxygen consumption rate

The oxygen consumption rate (OCR) of mBMECs was measured using the Seahorse Bioscience XF-96 Extracellular Flux Analyzer as previously described (Agilent, Santa Clara, CA) (Beeson et al., 2010). Cells were plated at a density of 1×10^4^ cells/well in 96-well plates. Each plate was treated with either vehicle (DMSO; <0.5%) or lasmiditan (0-100 nM) for 48 h in fetal bovine serum-free media. Basal OCR was measured before injection of carbonyl cyanide 4-(trifluoromethoxy) phenylhydrazone (FCCP; 2 μM; Sigma-Aldrich, St. Louis, MO) to measure uncoupled OCR, a marker of functional MB (Beeson et al., 2010).

### 2.4 Transmission electron microscopy

mBMECs were plated at a density of 5×10^4^ on D35 cell culture dishes and treated with vehicle or 3 nM lasmiditan for 24 h. mBMECs were then fixed and sectioned for transmission electron microscopy (TEM) as previously described (Dupre et al., 2019; Scholpa et al., 2019). Images were viewed using the FEI Tecnai Spirit microscope (FEI, Hillsboro, OR) operated at 100 kV and captured using an AMT 4 Mpixel camera (Advanced Microscopy Techniques, Woburn, MA) at a magnification of 16,500X. Mitochondrial count and morphology were analyzed using the analyze particles plug-in in ImageJ FIJI. In all cases, four to five images were analyzed blindly per sample.

### 2.5 Protein isolation and immunoblot analysis

Protein was extracted using RIPA buffer with protease inhibitor cocktail (1:100), 1 mM sodium fluoride, and 1 mM sodium orthovanadate (Sigma-Aldrich, St. Louis, MO) as described previously (Dupre et al., 2019; Scholpa et al., 2019). Protein was quantified using a bicinchoninic acid assay, and up to 10 μg of protein was separated via electrophoresis using 4–15% SDS-PAGE gels, then transferred to nitrocellulose membranes (Bio-Rad, Hercules, CA). Membranes were blocked in 5% milk or BSA in TBST and incubated overnight with primary antibodies with constant agitation at 4°C. Membranes were incubated with the appropriate horseradish peroxidase-conjugated secondary antibody and visualized using chemiluminescence (Thermo Scientific, Waltham, MA) on a GE ImageQuant LAS-4000 (GE Life Sciences, Pittsburg, PA). Optical density was determined using Image Studio Lite software. Primary antibodies used were PGC-1α (1 μg/mL; Abcam ab191838, Cambridge, MA), ATPSB (1 μg/mL; Abcam ab14730), CD31 (0.523 μg/mL; Abcam ab222783), claudin-5 (0.5 μg/mL; Invitrogen 35–2,500, Carlsbad, CA), ZO-1 (0.22 μg/mL; Abcam ab96587), occludin (0.71 μg/mL; Abcam ab216327), VE-cadherin (0.238 μg/mL; Abcam ab205336), p-VE-cadherin (Tyr658; 0.2 μg/mL; Invitrogen 44-1144G), p-Akt (Ser473; 0.091 μg/mL; Cell Signaling 9,271, Danvers, MA), Akt (0.031 μg/mL; Cell Signaling 9,272), p-eNOS (Ser1177; 0.485 μg/mL; Abcam ab215717), eNOS (0.25 μg/mL; Abcam ab76198), Nop-FoxO1 (Ser256; 0.107 μg/mL; Cell Signaling 9,461 s), FoxO1 (0.088 μg/mL, Cell Signaling 2,880 s), and tubulin (0.065 μg/mL, Abcam ab52866).

### 2.6 Cellular migration assay

Migration was assessed and analyzed as previously described (Liang et al., 2007; Dupre et al., 2019) via MRI wound healing plug-in in ImageJ FIJI. Briefly, mBMEC were plated at a density of 5×10^4^ on D35 cell culture dishes and grown to confluence. Cells were then treated with either vehicle or 3 nM lasmiditan for 24 h in serum-free media. Scratches were applied using a 10-μL pipette tip, and media were replaced with complete media containing lasmiditan or vehicle. Images of five randomly selected areas of the scratch were taken for each treatment at each time point. The percent migration was determined by the following formula: % area of migration 6 h post-scratch = (scratch area 0 h post-scratch − scratch area 6 h post-scratch) / (scratch area 0 h post-scratch) * 100.

### 2.7 Tube formation assay

mBMEC were plated at a density of 2.2×10^6^ on D100 cell culture dishes pretreated with Gibco attachment factor and grown to approximately 80% confluence in endothelial cell media. Cells were then treated with either vehicle or 3 nM lasmiditan for 24 h. After 24 h, mBMEC were collected via Accutase dissociation and centrifuged at 300 x *g* for 10 min at 4°C to form a pellet. Cells were resuspended in endothelial cell media containing either vehicle or lasmiditan, counted, and diluted to 1.5×10^5^ cells/mL, and 50 μL of cell suspension was added to an Ibidi (Fitchburg, WI) μ-Slide 15 Well 3D cell culture slide precoated with 10 μL of Corning reduced growth factor phenol red free Matrigel solution (Corning, NY), as per manufacturer’s recommended protocol. mBMEC were allowed to grow in Matrigel-coated wells for 24 h, and wells were imaged with a 10x LWD objective on an EVOS M5000 microscope under phase contrast with a light diffuser insert to help prevent uneven illumination.

Ten images from each lot/passage were analyzed using Ibidi FastTrack AI software (provided by MetaVi Labs).

### 2.8 TEER

mBMEC were plated at a density of 1.8×10^5^ cells/well on polyester-coated Transwell inserts (CLS3460; Corning) of 0.4 μm pore size in endothelial cell media and grown to confluence. For monoculture experiments, membranes were then placed in 12-well plates containing endothelial cell media and treated with 3 nM lasmiditan or vehicle. For co-culture experiments, 2×10^5^ astrocytes/well were plated and grown 48 h in 12-well plates with DMEM. Inserts containing mBMEC were then added to the astrocyte-containing wells and treated with 3 nM lasmiditan or vehicle in endothelial cell media. TEER was assessed using an EVOM^2^ Epithelial Voltohmmeter with chopsticks electrode set (World Precision Instruments) after 24 h (day 1) and again after 48 h (day 2). Net resistance (Ω) was determined by subtracting that of media-only blank wells, and TEER was calculated based on the area of the well as Ω·cm^2^ (Srinivasan et al., 2015).

### 2.9 Statistical analysis

Primary mBMEC isolated from a single passage represents an individual experiment (*n* = 1) and *n* = 5-6/group was used for each experiment. Data were normalized to respective vehicle-treated samples. Differences between two groups were analyzed using a two-tailed paired *t*-test, while that of three or more groups was analyzed using a one-way ANOVA followed by Tukey’s *post-hoc* test. In all cases, GraphPad Prism software (La Jolla, CA) was used and a *p* < 0.05 was considered statistically different between mean values.

## 3 Results

### 3.1 5-HT_1F_R agonism induces mitochondrial biogenesis in mBMEC

**Figure 1:**
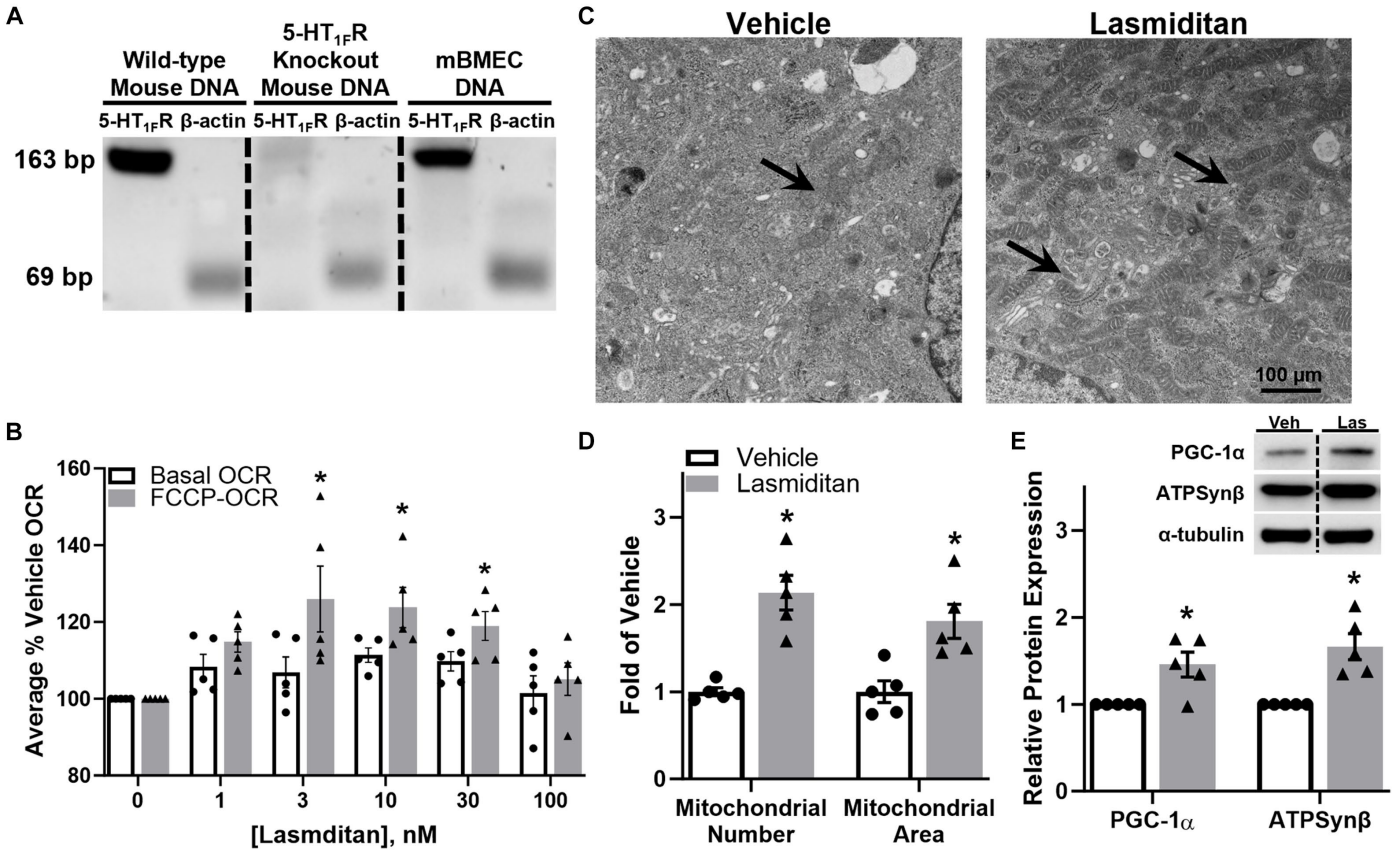
Effect of lasmiditan on mitochondrial biogenesis in mBMEC. The presence of the 5-HT_1F_ receptor was confirmed in mBMEC via PCR **(A)**. mBMEC were treated with either vehicle or lasmiditan (1–300 nM) for 48 h. Uncoupled mitochondrial oxygen consumption rates (FCCP-OCR) were measured using a Seahorse Bioscience XF-96 analyzer **(B)**. Based on these data, mBMEC were treated with vehicle or 3 nM lasmiditan for 72 h and either assessed via TEM (**(C)**; scale bar = 100 μm) to determine mitochondrial number and area **(D)** or analyzed for MB-related proteins **(E)**. Data represent *n* = 5 and are expressed as mean ± SEM (**(B)** *p < 0.05 compared to vehicle using one-way ANOVA followed by Dunnett’s *post-hoc* test; **(D,E)** *p < 0.05 compared to vehicle using a paired *t*-test).

Prior to experimentation, expression of the 5-HT_1F_R was confirmed in mBMEC via PCR (Figure 1A). FCCP-uncoupled oxygen consumption rate (OCR) is a marker of maximal ETC activity and potential MB (Beeson et al., 2010; Wills et al., 2012; Garrett et al., 2014; Cleveland et al., 2020; Simmons et al., 2021). mBMEC were exposed to various concentrations of lasmiditan for 48 h and then assessed for FCCP-OCR. Lasmiditan had a concentration-dependent effect on FCCP-OCR, with a ~ 25% increase with a 3 nM, the lowest effective concentration (Figure 1B). Based on this, 3 nM lasmiditan was used for all experiments. Transmission electron microscopy (TEM) was used to assess mitochondrial content in mBMEC (Figure 1C). Following lasmiditan exposure, mBMEC exhibited increased mitochondrial number and area (Figure 1D) per field compared to vehicle controls, further indicating MB. Immunoblot analysis revealed increased PGC-1α, the master regulator of MB, and ATP synthase β (ATPSB), a subunit of the ETC, with lasmiditan treatment compared to vehicle (Figure 1E).

### 3.2 5-HT_1F_R agonism stimulates migration in mBMEC

**Figure 2:**
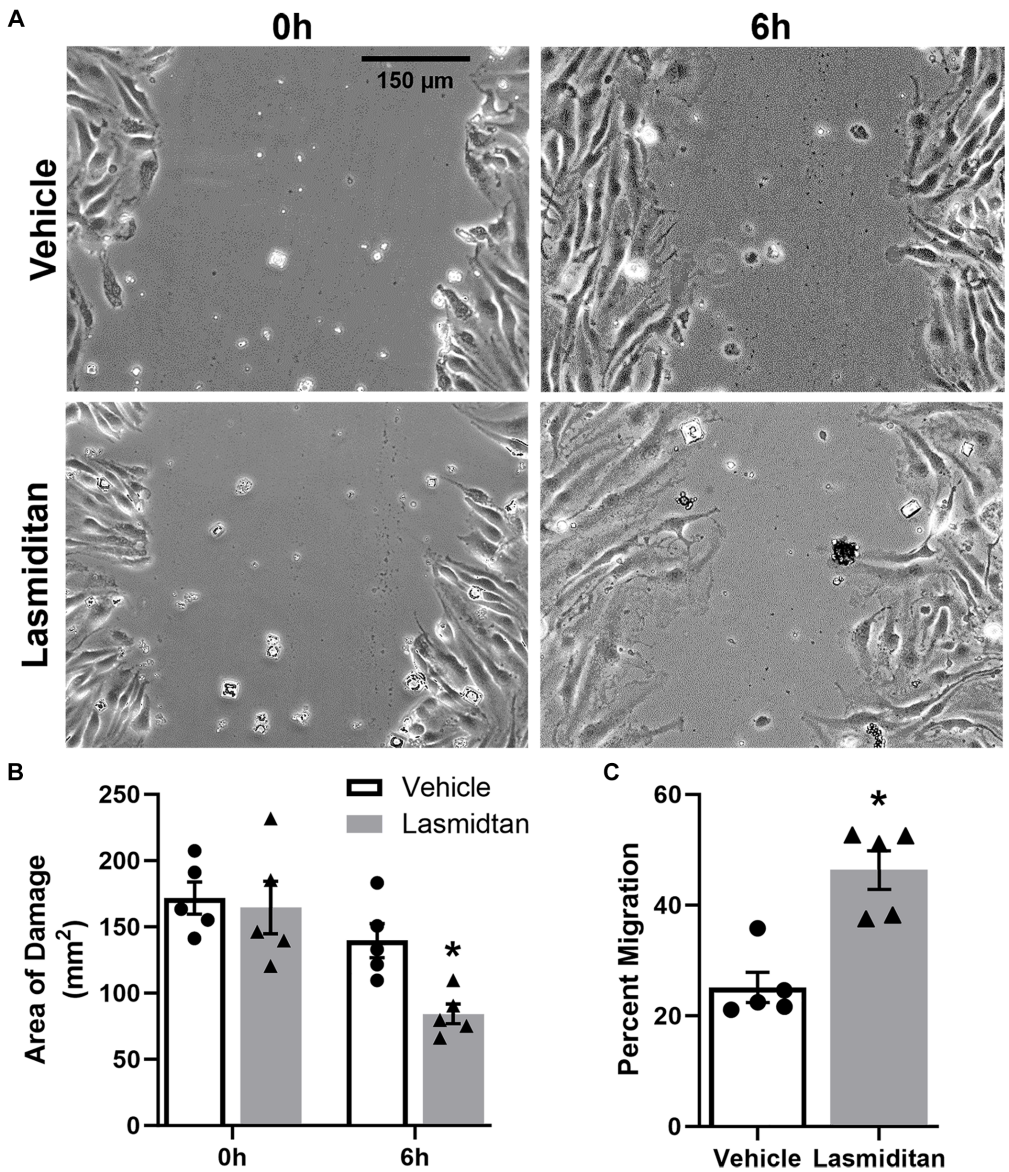
Effect of lasmiditan on cell migration in mBMEC. mBMEC were grown to confluence and then treated with vehicle or 3 nM lasmiditan for 24 h in serum-free media. The cell monolayer was scratched with a 10-μL pipette tip, and serum-free media were replaced with complete media containing lasmiditan or vehicle. mBMEC were imaged at 0 h and 6 h post-injury (**(A)**; scale bar = 150 μm) to determine the area of damage **(B)** and the percent of migration **(C)**. Data represent *n* = 5 and are expressed as mean ± SEM (*p < 0.05 compared to vehicle using a paired *t*-test).

A wound healing model was used to determine whether lasmiditan stimulates migration in mBMEC (Liang et al., 2007) (Figure 2A). Cells were grown to confluence and treated with vehicle or 3 nM lasmiditan for 24 h in serum-free media, after which a scratch was applied (0 h). Lasmiditan treatment decreased the area of damage by ~60% compared to vehicle control by 6 h (Figure 2B), equating to a 2-fold increase in cell migration (Figure 2C). Importantly, no difference in wound area was observed upon injury.

### 3.3 5-HT_1F_R agonism stimulates tube formation in mBMEC

**Figure 3:**
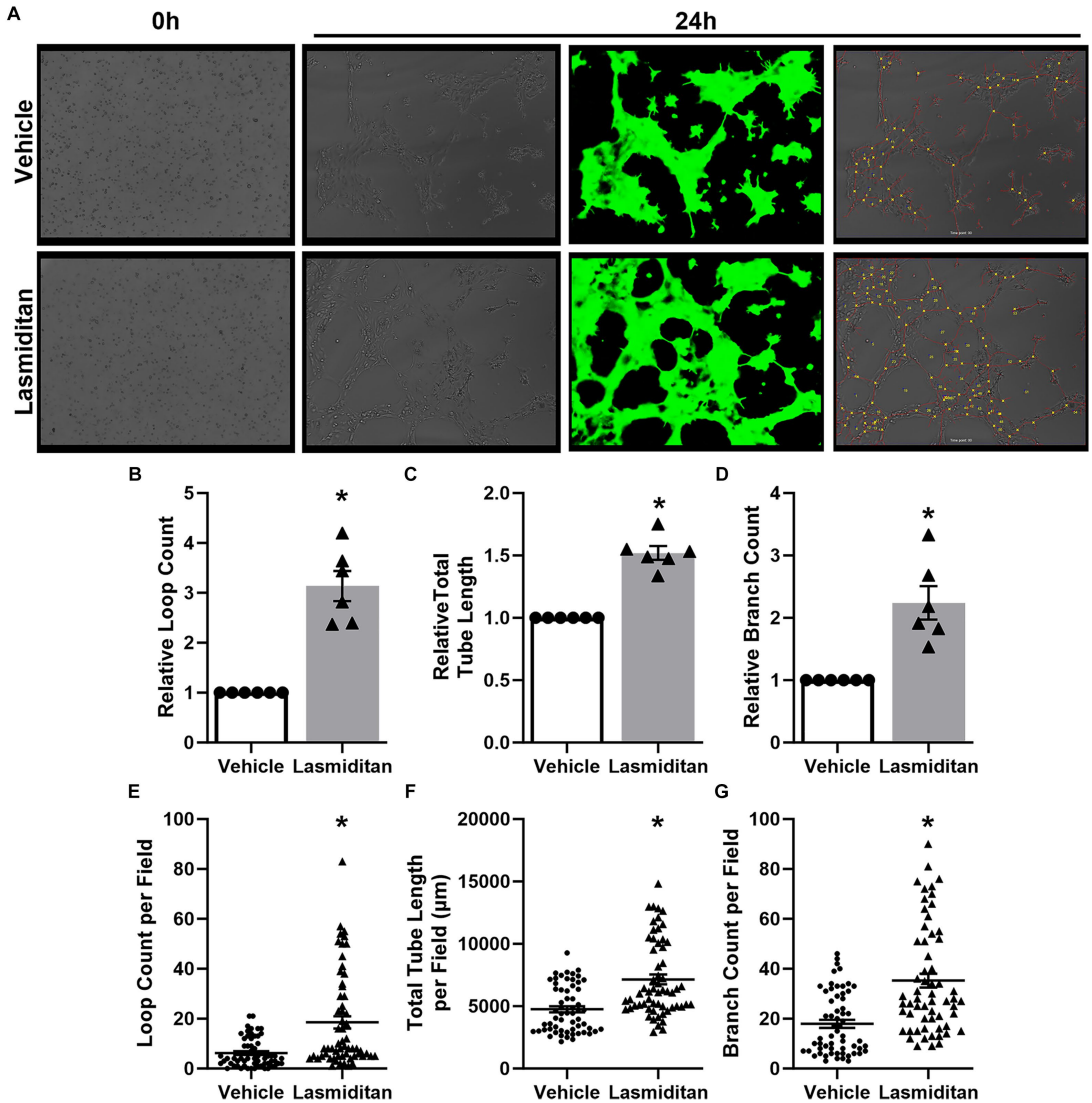
Effect of lasmiditan on tube formation in mBMEC. mBMEC were grown to ~80% confluence and then treated with vehicle or 3 nM lasmiditan for 24 h. Cells were then collected and added to wells of an ibdi μ-Slide 15 Well 3D cell culture slide precoated with 10 μL of Corning reduced growth factor phenol red free Matrigel solution at a density of 7.5×10^3^ cells/well (0 h) where they were allowed to grow for an additional 24 h in the presence of vehicle or lasmiditan **(A)**. Ten images per lot/passage were collected, and Ibidi FastTrack AI software (provided by MetaVi Labs) was used to apply a pseudo-casein tracking mask (green) and to determine loop count **(B,E)**, total tube length **(C,F)**, and branch count **(D,G)**. For **(B–D)**, data are normalized to the respective vehicles and represent *n* = 6/group. **(E–G)** Depict raw data collected from each image, representing 60 images per group. Data are expressed as mean ± SEM (*p < 0.05 compared to vehicle using a paired *t*-test).

A Matrigel-based tube formation assay was used to assess the angiogenic potential of lasmiditan in mBMEC (Arnaoutova and Kleinman, 2010; Almeria et al., 2019; Hunter et al., 2022) (Figure 3A). Following 24 h of treatment, mBMEC were added to Matrigel-coated wells (0 h) and allowed to grow for an additional 24 h in the continued presence of vehicle or 3 nM lasmiditan. Lasmiditan treatment increased loop count 3-fold (Figure 3B), total tube length 1.5-fold (Figure 3C), and branch count 2-fold (Figure 3D) in mBMEC. Figures 3E–G depict the loop count, total tube length, and branch count for all images collected.

### 3.4 5-HT_1F_R agonism increases electrical resistance in mBMEC

**Figure 4:**
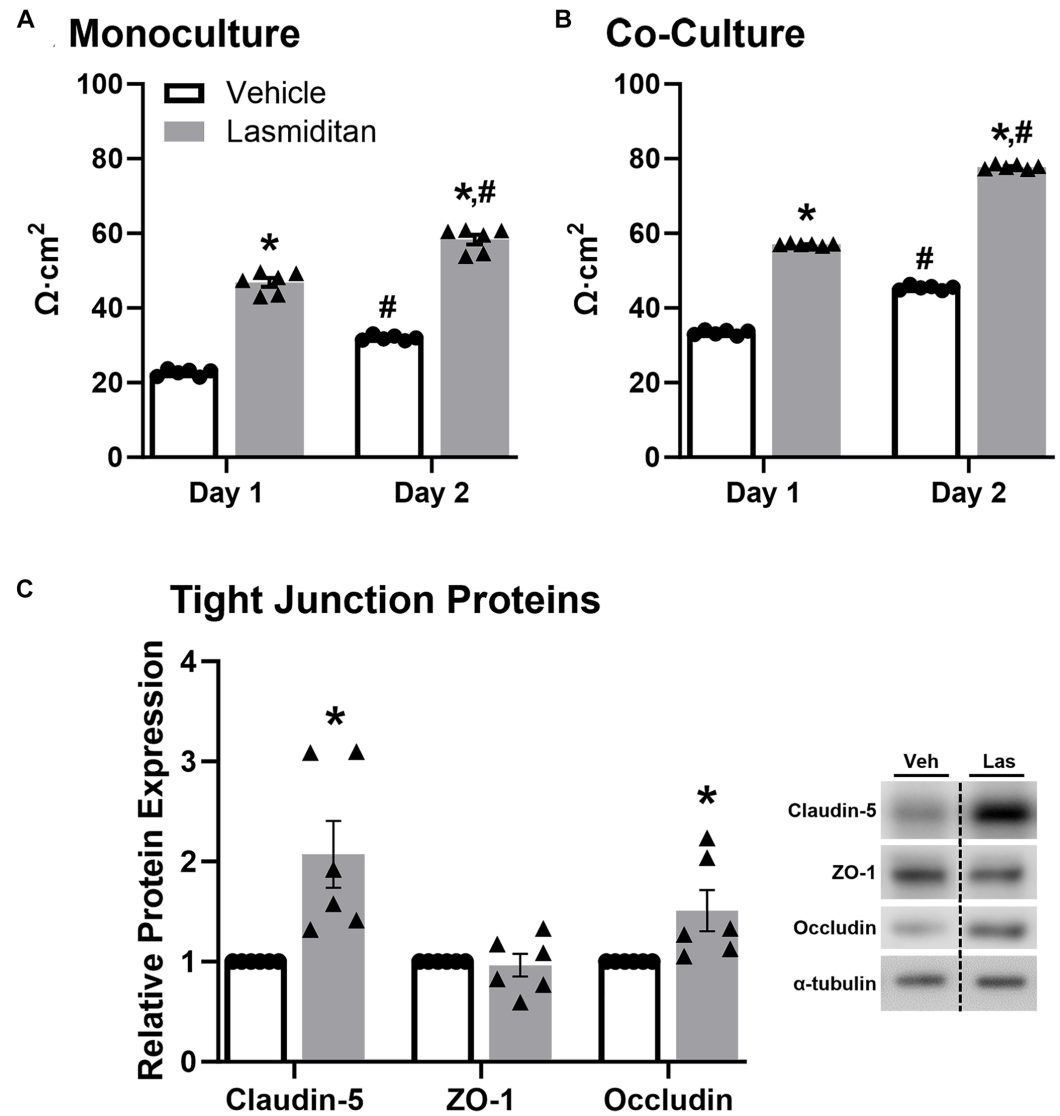
Effect of lasmiditan on trans-endothelial electrical resistance in mBMEC. mBMEC were plated on polyester-coated transwell inserts at a density of 1.8×10^5^ and grown for 48 h. Membranes were placed in 12-well plates containing either endothelial cell media **(A)** or astrocytes **(B)** and then treated with vehicle or 3 nM lasmiditan. TEER was measured after 24 h (day 1) and 48 h (day 2). Protein was isolated from the mBMEC from the co-culture TEER condition, and the expression of tight junction proteins was assessed **(C)**. Data represent *n* = 6 and are expressed as mean ± SEM (*p < 0.05 compared to vehicle, #*p* < 0.05 compared to day 1 using a paired *t*-test).

Trans-endothelial electrical resistance (TEER) was used to examine the effect of lasmiditan on mBMEC barrier function with and without astrocyte co-culture. When grown in monoculture, mBMEC TEER increased nearly 2-fold when treated with lasmiditan compared to vehicle on both day 1 (43 v 24 Ω·cm^2^) and day 2 (58 v 31 Ω·cm^2^) (Figure 4A). Similar results were seen in the presence of astrocytes, where lasmiditan treatment increased mBMEC TEER from 33 to 57 Ω·cm^2^ on day 1 and from 45 to 78 Ω·cm^2^ on day 2 (Figure 4B). Regardless of the presence or absence of astrocytes or lasmiditan, mBMEC TEER increased from day 1 to day 2. As expected, TEER values were inherently higher with astrocyte co-culture (Abbott, 2002); however, these data suggest that astrocytes have no effect on the lasmiditan-induced increase in mBMEC barrier integrity. Protein was collected from the mBMEC that had undergone TEER assessment in the presence of astrocytes (Figure 4B), and the expression of tight junction proteins was assessed. Claudin-5 and occludin increased 2- and 1.5-fold with lasmiditan treatment, respectively, while no effect was observed with ZO-1 (Figure 4C).

### 3.5 5-HT_1F_R agonism decreases VE-cadherin phosphorylation and activates the VE-cadherin–Akt–FoxO1 signaling axis in mBMEC

**Figure 5:**
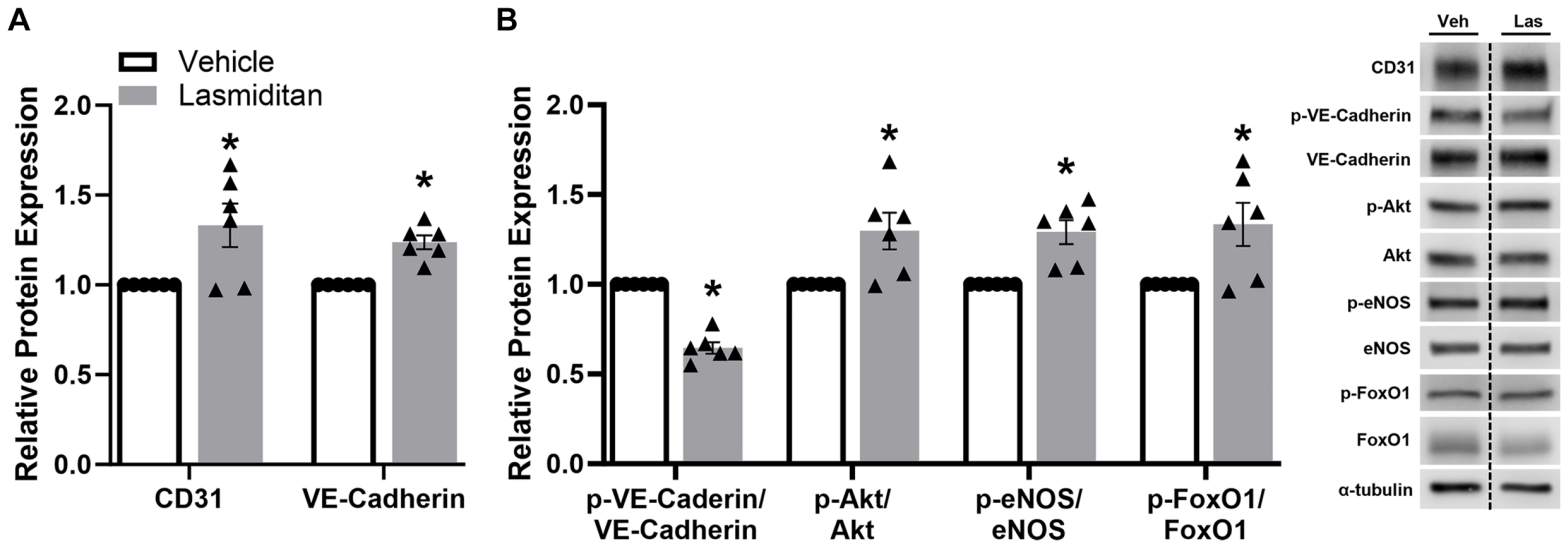
Effect of lasmiditan on the VE-cadherin–Akt–FoxO1 signaling axis in mBMEC. mBMEC were plated on 6-well plates at 3×10^5^ cells/well, grown for 24 h, and then treated with vehicle or 3 nM lasmiditan for 48 h. Cells were collected and the expression of endothelial cell-related proteins (A) and members of the VE-Cadherin-Akt-FoxO1 signaling axis (B) were assessed. Data represent *n* = 6 and are expressed as mean ± SEM (*p < 0.05 compared to vehicle using a paired *t*-test).

To begin to discern the pathway of the effect of lasmiditan on endothelial cell function in mBMEC, cells were exposed to lasmiditan or vehicle for 48 h, and protein was isolated for immunoblot analysis. Vascular markers CD31 and VE-cadherin were increased in the lasmiditan-treated compared to vehicle-treated cells (Figure 5A). Additionally, phosphorylation of VE-cadherin at Tyr658, which has been found to disrupt VE-cadherin-mediated junctions (Potter et al., 2005), was decreased with lasmiditan treatment. VE-cadherin can directly contribute to the expression of claudin-5, in that adhesion triggers phosphorylation of Akt and, subsequently, phosphorylation of FoxO1, which prevents its translocation into the nucleus where it can repress claudin-5 transcription (Gavard and Gutkind, 2008). Importantly, lasmiditan-treated mBMEC exhibited increased Akt, eNOS, and FoxO1 phosphorylation compared to vehicle-treated cells (Figure 5B).

## 4 Discussion

Disruption of mitochondrial quality control mechanisms is implicated in the onset and progression of many CNS disorders. In addition to impaired cellular processes and the initiation of cell death mechanisms, mitochondrial dysfunction also impacts vascular integrity, further propagating disease (Vlodavsky et al., 2017). Therefore, targeting the restoration of mitochondrial homeostasis and thereby improving vascular integrity could prove a valuable strategy to promote recovery from neuropathologies. Existing data demonstrate that restoring mitochondrial homeostasis via pharmacological stimulation of MB can promote recovery after CNS injury or neurodegenerative disease (Wang et al., 2015; Li et al., 2016; Golpich et al., 2017; Wang et al., 2019). Our group previously reported that agonism of the 5-HT_1F_R is a potent inducer of MB peripherally and centrally (Gibbs et al., 2018a; Scholpa et al., 2018; Dupre et al., 2019; Simmons et al., 2020a, 2021; Hurtado et al., 2023a,b). Furthermore, pharmacological induction of MB via 5-HT_1F_R agonism improved recovery following traumatic SCI and in a mouse model of Parkinson’s disease (Scholpa et al., 2018; Simmons et al., 2020a, 2021). Here, we report treatment with the FDA-approved selective 5-HT_1F_R agonist lasmiditan induces MB, increases migration, promotes angiogenesis, and enhances vascular integrity in CNS endothelial cells.

While endothelial cells primarily utilize aerobic glycolysis as their source of energy production (Caja and Enríquez, 2017), we observed that lasmiditan stimulated mBMEC oxidative respiration as evidenced by an increase in FCCP-OCR. Similar to observations reported in renal endothelial cells (Dupre et al., 2019), mBMEC treated with lasmiditan exhibited increased maximal mitochondrial respiration, mitochondrial density and area, and PGC-1α, the master regulator of MB. Reports demonstrate that pharmacological activation of MB promotes angiogenesis and restoration of barrier integrity, associated with improved pathological and behavioral outcomes following CNS trauma (Simmons et al., 2020b). Additionally, PGC-1α is documented to stimulate angiogenesis in endothelial cells (Mammoto et al., 2018). In support of this, lasmiditan-treated mBMEC exhibited enhanced tube formation and wound closure following scratch injury, *in vitro* models established to mimic key components of angiogenesis *in vivo* (Lamalice et al., 2007; Liang et al., 2007; Arnaoutova and Kleinman, 2010). Blood–CNS barrier integrity and permeability can be evaluated by measuring the electrical resistance of a cell monolayer (Srinivasan et al., 2015). We report increased TEER in mBMEC treated with lasmiditan, with and without co-culture with astrocytes, suggesting enhanced barrier integrity and further supporting increased endothelial cell function with 5-HT_1F_R agonism.

To maintain vascular integrity, it is critical for new and recovering vessels of blood–CNS barriers to stabilize after angiogenesis. Cell adhesion and tight junction proteins play integral roles in this stability. We observed increased tight junction protein expression as well as increased VE-cadherin, a key cell adhesion molecule responsible for maintaining endothelial barrier function and angiogenesis (Giannotta et al., 2013), in mBMEC treated with lasmiditan. Additionally, lasmiditan treatment decreased phosphorylation of VE-cadherin at Tyr658, which has been shown to decrease adhesion and result in VE-cadherin internalization and, ultimately, junctional breakdown (Schimmel and Gordon, 2018). Therefore, in addition to increased VE-cadherin, the observed decrease in phosphorylation likely results in enhanced VE-cadherin adhesion.

Activation of the PI3K-Akt-eNOS pathway is considered a central factor in angiogenesis and cell migration (Lamalice et al., 2007), while also having implications in MB (Gibbs et al., 2018b,c; Simmons et al., 2020b). VE-cadherin adhesion triggers activation of this pathway, phosphorylation of Akt, and, subsequently, phosphorylation of FoxO1. Phosphorylation prevents the translocation of FoxO1 from the cytoplasm to the nucleus, where it can repress claudin-5 transcription (Gavard and Gutkind, 2008; Farhan et al., 2017). Therefore, reduced phosphorylation of VE-cadherin, and thereby increased adhesion and continued activation of Akt-eNOS-FoxO1, likely contributes to the increased levels of claudin-5 and improved barrier integrity observed in lasmiditan-treated mBMECs. Interestingly, phosphorylation of FoxO1 has also been shown to increase HIF-1 and VEGF expression and promote angiogenesis in some cancer models (Farhan et al., 2017), further implicating its involvement in endothelial cell function.

The results presented here not only corroborate existing data detailing a positive relationship between MB and angiogenesis, but also demonstrate 5-HT_1F_R agonist-induced enhancement of endothelial cell function, including angiogenesis, migration, and barrier integrity. Furthermore, these data suggest that this effect is likely due in part to increased VE-cadherin adhesion leading to FoxO1 phosphorylation and increased claudin-5 transcription. These findings support lasmiditan and 5-HT_1F_R agonism as promising potential therapeutic strategies for the treatment of various CNS pathologies such as SCI, TBI, and neurodegenerative diseases, through the promotion of MB, angiogenesis, and vascular recovery.

## Data availability statement

The raw data supporting the conclusions of this article will be made available by the authors, without undue reservation.

## Ethics statement

Ethical approval was not required for the studies on animals in accordance with the local legislation and institutional requirements because only commercially available established cell lines were used.

## Author contributions

NS: Conceptualization, Formal analysis, Investigation, Methodology, Supervision, Writing – original draft, Writing – review & editing, Funding acquisition, Project administration, Visualization. ES: Conceptualization, Investigation, Methodology, Writing – original draft, Writing – review & editing, Formal analysis, Funding acquisition, Project administration, Visualization. AT: Investigation, Methodology, Writing – original draft, Writing – review & editing, Formal analysis, Funding acquisition, Visualization. SC: Investigation, Writing – original draft, Writing – review & editing. RS: Conceptualization, Funding acquisition, Project administration, Resources, Supervision, Writing – original draft, Writing – review & editing.
